# Book review of applied medical image processing: A basic course

**DOI:** 10.1186/1475-925X-10-16

**Published:** 2011-02-27

**Authors:** Edward J Ciaccio

**Affiliations:** 1 Columbia University Medical Center, Harkness Pavilion 804, 180 Fort Washington Avenue, New York NY 10032

## Abstract

This article is a review of the book: 'Applied Medical Image Processing: A Basic Course', by Wolfgang Birkfellner, which is published by CRC Press. Basic information that should be helpful in deciding whether to read the book and whether to use it as a course textbook is presented. This includes an introduction, the suitability of the book for use in coursework, its coverage of medical imaging and image processing, discussion and conclusions, and an appendix with a relevant computer program for extracting medical images.

## Introduction

'Applied Medical Image Processing: A Basic Course' by Dr. Wolfgang Birkfellner is an outstanding work that will be of interest to virtually all biomedical engineers. Just published by CRC Press and in its first printing in 2011, it is not expensive - listed as $69.95 and available in hardcover. Although 403 pages in length, it is small in footprint and can readily be carried in the pocket of a book or computer bag. The text is easy to follow, supposing only that the reader has a modest knowledge of linear algebra, basic engineering principles, and computer programming.

I was impressed by the content as well as by the amount of information that is packed on each page. In each well-organized chapter, important concepts and definitions are described concisely and well. Relevant medical images used for processing compliment many of the concepts discussed. Where important, the mathematical formulation is provided. The text and images refer to several MATLAB-based programming examples that are provided at the end of each chapter. These are useful to apply the concepts to actual medical image processing. The book can be used in part as an excellent reference work for biomedical engineers, scientists, and clinicians.

## Suitability as a Course Textbook

This is a well-written, coherent, and comprehensive work covering the major topics in the field, suitable as a standalone text for coursework in medical image processing. It will mainly be of interest to course directors as an upper-level undergraduate or beginning graduate main textbook. For teaching upper-level undergraduates, the concepts presented can be supplemented with additional programming examples. For teaching beginning graduate students, the text should be supplemented with additional theoretical background in the lectures, as well as more challenging programming examples. For medical residents, lectures in the fundamental mathematics, engineering, and computer language principles may be needed prior to coverage of the image processing chapters. In Chapters 1, 2, and 9, medical imaging topics are presented which require less quantitative background information, while Chapters 3-8 detail specific aspects of medical image processing. Students with adequate preparation can be expected to understand the mathematical concepts after a careful examination of these chapters, without the necessity of gleaning supplemental information from other texts. The book coverage will provide the student with sufficient information to implement the concepts computationally. For many topics, tested example programs are provided in MATLAB code. Therefore, course lectures can emphasize the application of theoretical concepts to real-world medical image processing problems.

## Content of the Book

Applied Medical Image Processing should prove useful for learning the basics of this important field, and to refresh the memory of those who already have some background in the field. Included are a helpful Table of Contents, List of Figures and Tables, and a comprehensive Index. A compact disk containing all MATLAB programs referred to in the text is included in the inside back cover. Although most chapters are written by Dr. Birkfellner, Chapter 1 is written by Dr. Johann Hummel and Chapter 9 is written by Dr. Michael Figl.

Medical image sources are discussed in Chapter 1. This includes an emphasis on computed tomography (CT) - with descriptions of how images are generated and the types of image artifacts that can occur, and magnetic resonance imaging (MRI), including biophysical principles and elements of an MR system. The chapter continues with a discussion on ultrasound, nuclear medicine techniques, and good safety practices. In Chapter 2, image representation is presented, including the basics of pixels and voxels, grayscale and color representation, and commonly used medical image file formats such as DICOM. A discussion of image quality and signal-to-noise ratio follows.

The first of the image processing chapters - Chapter 3 - describes mathematical operations in intensity space, which are used to enhance medical images for improved visualization. The topics include the use of the intensity transfer function, how to prevent image saturation, the dynamic range of the imaging system, and use of windowing functions and histograms to improve grayscale contrast.

Digital filters as they are used to alter images in ways affecting computational analyses, and to extract salient information, are covered in Chapter 4. Additionally, mathematical transforms are introduced in this chapter, with transformational methods being presented to represent data for ease of measurement and to resolve differences between image classes.

Basic medical image segmentation methods for delineation of regions of interest (ROI) are introduced in Chapter 5. The topics include defining the ROI, methods for establishing ROI location, and centroid calculation. Implementation of techniques for ROI boundary detection and reduction of error in boundary definition are discussed. Lastly, performance indices to evaluate the results of segmentation are presented.

Spatial transforms for projecting three-dimensional volume data into two-dimensional images are described in Chapter 6. Very useful equations for two- and three-dimensional data translation and rotation are provided in a form that can readily be implemented computationally. Methods for interpolation of transformed data to reduce artifact during image construction are presented. The chapter also includes an extensive section on ROI tracking during patient diagnosis and treatment.

Quantitative image registration methods are covered in Chapter 7. First an explanation of fiduciary markers to registrate two or more images is discussed. Comparisons are then made between intramodal versus intermodal, and rigid versus nonrigid registration methods. The use of merit functions as measures of image similarity during the registration process is introduced. Lastly, strategies for local versus global optimization for image registration are considered, and evaluation of the results using target and fiducial registration error measurements are presented.

Implementation of rendering algorithms to construct two-dimensional images from a three-dimensional volume is the subject of Chapter 8. The methods that are emphasized include the use of windowing functions to enable information hidden in the full range of image intensities to be made visible, and reformatting so that the data cube is sliced along the desired viewing plane.

Construction of CT images is the subject of Chapter 9. X-ray attenuation during CT scanning, and reconstruction of the tissue density distribution is described by the Radon Transform, which is covered extensively in this chapter. Algebraic reconstruction of tissue regions from CT data is covered, including a discussion of methods to solve a system of linear equations. For faster computation, the filtered backprojection method is also discussed.

Rounding out the book are useful descriptions of commonly-used MATLAB commands in Chapter 10. These commands are incorporated in the main MATLAB program and do not require purchase of MATLAB toolboxes.

## Minor Criticisms

I have a few minor criticisms of the book. There are some typos in the first printing; however as the author mentions these can be reported to the publisher. Additionally, as the author notes, the text lacks color images. This is probably justified, as medical imaging and image processing are often done in grayscale, and the lack of color images reduces the book cost to financially burdened students and others. Where relevant, color images are provided in the compact disk. Another issue is that the MATLAB code presented in the examples at the end of most chapters is an interpreted computer language, which lengthens the program runtime. However this is helpful for learning, because program changes can readily be made and tested. For fast computation, programmers should implement their image processing steps in a compiled, high-performance computer language such as C or Fortran. Finally, although the treatment of medical image processing in this book is excellent, medical images are often acquired in the form of a videoclip, for example during capsule endoscopy of the small intestine. A program from my own work which extracts such images for image processing is provided in the Appendix of this review.

## Discussion and Conclusions

Applied Medical Image Processing: A Basic Course will be very helpful for understanding medical imaging and image processing, and easy reading for those with an engineering and computer programming background. For such readers it will not be hard to cover several chapters rapidly and to understand many important concepts, although implementation of the MATLAB code will require extra time. One can read the entire book and gain a good theoretical understanding while skipping the programming examples. As part of the theoretical treatment, the mathematical equations included in the text should not be overlooked as they are intrinsic to learning the concepts. The programming examples provide a practical understanding for implementation of medical image processing techniques.

Overall, I was impressed by the amount of information that can be learned, or relearned, on first reading of Applied Medical Image Processing. A quick second reading will serve to reinforce any details missed the first time around. As a computational biologist, I chose to read the chapters out of order according to my interests. In doing so, for continuity one may need to refer back to earlier parts of the book.

In conclusion, I would recommend Applied Medical Image Processing: A Basic Course, to engineers (biomedical and others), scientists, and clinicians. The book is enjoyable to read, and students and professionals will not be bored when doing so. Engineers and scientists will find useful coverage of the field, and students will find the entire volume to be filled with important information. Dr. Birkfellner and his colleagues are to be heartily congratulated for writing an edifying work that is sure to be useful and frequently used.

## Competing interests

The author declares that he has no competing interests.

## Appendix

The following program plays a color mpeg videoclip, and then stores the individual frames as 256-level grayscale pgm images in ASCII, which is a simple format to work with for image processing. Reading of videoclips in other formats, and writing to other image formats and to a binary file, is readily done by changing a few of the parameters. The play speed for the movie, 2/s for my application, is altered via the last parameter in the movie function and can be used to slow down or speed up play. The code was implemented in MATLAB ver. 7.7.0, R2008b (The MathWorks, Natick, MA), the same version as is used in the book. As with the book examples, the program below requires only the main MATLAB program to run.

% Construct an object associated with the mpeg videoclip.

readerobj = mmreader('C:\folder\filename.mpg', 'tag', 'myreader1');

% Read in all video frames and obtain the number of frames.

vidFrames = read(readerobj);

numFrames = get(readerobj, 'numberOfFrames');

% Create a MATLAB movie structure from the video frames.

for k = 1 : numFrames

mov(k).cdata = vidFrames(:,:,:,k);

mov(k).colormap = [ ];

end

% Create figure, resize from video dimensions, playback at frame rate (2/s).

hf = figure;

set(hf, 'position', [150 150 readerobj.Width readerobj.Height]);

movie(hf, mov, 1, 2);

% Write movie frames to individual grayscale files (pgm is simple format).

for k = 1 : numFrames

imwrite(mov(k).cdata, ['C:\folder\im' num2str(k, '%.3d') '.pgm'], 'pgm', 'Encoding', 'ASCII');

end

